# Assess the expression of ubiquitin specific protease *USP2a* for bladder cancer diagnosis

**DOI:** 10.1186/s12894-015-0074-x

**Published:** 2015-08-07

**Authors:** Pildu Jeong, Yun-Sok Ha, Seok-Joong Yun, Hyung Yoon Yoon, Michael R. Freeman, Jayoung Kim, Wun-Jae Kim

**Affiliations:** Department of Urology, Chungbuk National University College of Medicine, Cheongju, Chungbuk South Korea; Departments of Surgery, Harvard Medical School, Boston, 02115, MA USA; Cancer Biology Division, Departments of Surgery and Biomedical Sciences, Cedars-Sinai Medical Center, Los Angeles, CA 90048 USA

## Abstract

**Background:**

Given that a deubiquitinating enzyme, ubiquitin-specific protease 2a (USP2a), regulates ubiquitination, trafficking, and degradation of EGFR, which plays a critical role in bladder cancer, in this study, we aimed to quantify the USP2a gene expression, and to determine the possibility that USP2a can be used for bladder cancer diagnosis.

**Methods:**

Using two independent cohorts (cohort 1, *n* = 339 in total; cohort 2, *n* = 140 in total) consisting of human bladder tissues from BC patients and normal controls, we analyzed the gene expression levels of *USP2a.* A quantitative real-time PCR amplification was performed using a Rotor Gene 6000 instrument to quantify the expression of *USP2a* mRNA.

**Results:**

A comparison of 305 bladder cancers and 34 age-matched controls showed an 81.4 % reduction in *USP2a* expression in bladder cancers as compared to normal bladder tissues (*p* < 0.001). In the independent cohort consisting of 140 BC tissues and matched adjacent normal bladder tissues, the levels of *USP2a* in the specimens of BC patients were reduced by 86.9 % as compared to matched surrounding normal specimens from the same patients (*p* < 0.001). Furthermore, there was 36.3 % reduction of *USP2a* gene expression in muscle invasive bladder cancer (MIBC, *n* = 121), compared to non muscle invasive bladder cancer (NMIBC, *n* = 184) (*p* = 0.004). Lastly, *USP2a* mRNA expression was significantly reduced in higher stages of MIBC patients (*p* = 0.024), but not in NMIBC patients.

**Conclusions:**

Our findings suggest that *USP2a* mRNA may be considered as a diagnostic marker candidate for bladder cancer, in particular, to stratify MIBC patients with a more invasive phenotype.

## Background

Bladder cancer (BC) is the second most common genitourinary malignancy and the fourth most common cancer among American and European [1–4]. More than 90 % of bladder cancers diagnosed in the US are urothelial carcinoma, which are mostly papillary, well-, or moderately-differentiated non-muscle invasive bladder cancer (NMIBC) [5]. Within 2 years after standard treatment, approximately 20–30 % of the NMIBC patients will show recurrence that results in the progression to muscle invasive disease (MIBC) [5], resulting in poor long-term survival and frequent metastases into regional lymph nodes (78 %), liver (38 %), lung (36 %), bone, adrenal gland or intestine [6].

A promising finding by a research team led by François Radvanyi, along with previous studies from this group and from other laboratories [7–9], provided persuasive evidence that a certain subtype of BC—“basal-like BC”—displays activation of EGFR signaling pathway and better responsiveness to EGFR inhibitors. EGFR is known to be de-ubiqutunated by ubiquitin-specific protease 2a (USP2a) [10]. In particular, in BC cell culture system, USP2a de-ubiquitinates a cell cycle regulator, cyclin A1, thereby promoting cell proliferation [11], and USP2a regulates expression levels of fatty acid synthase (FASN), MDM2, MDM4, MDMX, and cyclin D1 [12–16]. However, USP2a has been also known to have a distinct function as a signal mediator of tumor necrosis factor-α (TNF-α)-induced cell death [17], suggesting that USP2a function can be different in terms of cell context. Therefore, we hypothesized that USP2a expression correlates with bladder cancer progression and tested the hypothesis using our two unique cohorts consisting of patient-derived NMIBC and MIBC bladder tumor specimens.

## Methods

### Ethics Statement

The Ethics Committee of Chungbuk National University approved the protocol used for this study. Written informed consent was obtained from each subject. The Institutional Review Board of Chungbuk National University approved collection and analysis of all samples.

### Patients and Tissue Samples

All primary tumor samples from patients who underwent TUR or radical cystectomy were histologically verified as urothelial carcinoma at Chungbuk National University in South Korea [18, 19]. Non-cancerous surrounding tissue was obtained from tissue at least 3 cm from the tumor and normal bladder mucosa was harvested from patients with benign diseases such as BPH, ureter stone and stress urinary incontinence after informed consent. All control tissues were histologically confirmed as normal. Patients with concomitant carcinoma *in situ*, a short term follow-up period (less than 6 months), or for whom there was incomplete data collection were excluded in order to make the study population more homogeneous. The cohort 1 was consisted of 305 (253 male and 52 female with average age, 65 years old) BC patients and 34 controls (19 male and 15 female with average age, 54 years old), and the independent cohort 2 was consisted of 140 BC patients. All tumors were macro-dissected, typically within 15 min of surgical resection. Each bladder cancer specimen was confirmed by pathological analysis of a part of the tissue sample in fresh frozen sections from TUR or cystectomy specimens, and was then frozen in liquid nitrogen and stored at −80 °C until use. In the case of NMIBC, a second TUR was performed 2–4 weeks after the initial resection if a bladder cancer specimen did not include the proper muscle or when a high-grade tumor was detected. Patients who had a T1 tumor, multiple tumors, large tumors (>3 cm in diameter), or high grade Ta NMIBC received one cycle of intravesical treatment (e.g., 6-weekly treatment of BCG or 8-weekly treatment of mitomycin-C). Response to treatment was assessed by cystoscopy and urinary cytology. Patients who were free of disease within 3 months after treatment were assessed every 3 months for the first 2 years and then every 6 months thereafter. In the case of MIBC, patients with clinically localized or locally advanced tumors and good ECOG performance status (0 or 1) underwent radical cystectomy and complete pelvic lymph node dissection using an extended lymphadenectomy. Patients who were not eligible for radical cystectomy due to metastatic disease, poor life expectancy, or poor ECOG performance status (≥2) underwent TUR or biopsy for histopathological diagnosis. Patients with pT3, pT4 or node-positive disease based on the analysis of radical cystectomy specimens, or with metastatic disease but good performance status, received at least four cycles of cisplatin-based chemotherapy. Patients who refused or did not complete an imaging work-up such as a CT scan or MRI at least once every 3 months to evaluate response also were excluded from analysis.

Tumors were staged and graded according to the 2002 TNM classification and the EAU guideline based on 1973 WHO grading system [20–22]. Recurrence was defined as recurrence of primary NMIBC with a lower or the same pathological stage, and progression was defined as disease with T2 and higher stage upon relapse in NMIBC. In case of MIBC, progression was defined as local regional recurrence or a new distant metastasis in the cystectomized group and a ≥ 20 % increase in the mass of the primary tumor or a new distant metastasis in the non-cystectomized group.

### RNA extraction and construction of cDNA

RNA was isolated from tissue using 1 ml of TRIzol (Invitrogen, Carlsbad, CA) with homogenization in a 5-ml glass tube. The homogenate was transferred to a 1.5-ml tube and then mixed with 200 ml of chloroform. After incubation for 5 min at 4 °C, the homogenate was centrifuged for 13 min at 13,000 g at 4 °C. The upper aqueous phase was transferred to a clean tube with 500 ml of isopropanol. The mixture was incubated for 60 min at 4 °C followed by centrifugation for 8 min at 13,000 g, 4 °C. The upper aqueous phase was discarded and mixed with 500 ml of 75 % ethanol, and centrifuged for 5 min at 13,000 g, 4 °C. The upper aqueous layer was discarded and the pellet was dried at room temperature, dissolved in DEPC-treated water, and then stored at −80 °C. The quality and integrity of the RNA were confirmed using Nanodrop. cDNA was prepared from 1 mg of total RNA using a First-Strand cDNA Synthesis Kit (Amersham Biosciences Europe GmbH, Freiburg, Germany) according to the manufacturer’s protocol.

### Real-time PCR

Real-time PCR amplification was performed using a Rotor Gene 6000 instrument (Corbett Research, Mortlake, Australia) to quantify the expression of *USP2a* mRNA. Real-time PCR assays were carried out in micro-reaction tubes (Corbett Research, Mortlake, Australia) using SYBR Premix EX Taq (TAKARA BIO INC., Otsu, Japan). The following primers were used to determine *USP2a* mRNA levels: forward 5′-TGCTGAGACCCGACATCACT-3′; reverse 5′-TGGGGTCTATCCGGTAGCTA-3′, as described in previous literature [12]. The PCR reaction was performed in a final volume of 10 ml consisting of 5 ml of 2 X SYBR premix EX Taq buffer, 0.5 ml each of 59- and 39- primer (10 pmol/ml), and 1 ml of the sample cDNA. The product was purified with a QIAquick Extraction kit (QIAGEN, Hilden, Germany), quantified with a spectrophotometer (Perkin Elmer MBA2000, Fremont, CA), and then sequenced with an automated laser fluorescence sequencer (ABI PRISM 3100 Genetic Analyzer, Foster City, WI). Ten-fold serial dilutions of a known concentration of the product (from 100 pg/ml to 0.1 pg/ml) were used to establish the standard curve for real-time PCR. The real-time PCR conditions were as follows: 1 cycle for 20 s (seconds) at 96 °C, followed by 40 cycles of 2 s at 96 °C for denaturation, 15 s at 60 °C for annealing, and 15 s at 72 °C for extension. The melting program was performed at 72–95 °C with a heating rate of 1 °C per 45 s. Spectral data were captured and analyzed using Rotor-Gene Real-Time Analysis Software 6.0 Build 14 (Corbett Research, Mortlake, Australia). All samples were run in triplicate. Glyceraldehyde-3-phosphate dehydrogenase (GAPDH) was analyzed as an endogenous RNA reference gene and gene expression was normalized to the expression of GAPDH.

### Statistical Analysis

Differences in continuous variables between groups were assessed by one-way ANOVA analysis.

## Results

### Clinical and pathological characteristics of patients with bladder cancer

A previous study from our laboratory presented evidence suggesting that the USP2a deubiquitinase plays a role in bladder cancer cell proliferation using a cell culture system [11]. In order to approach the question of whether USP2a is involved in bladder cancer progression and aggressiveness, we analyzed *USP2a* mRNA expression level by qRT-PCR using human specimens from 305 patients with bladder cancer and 34 age-matched controls. Expression levels were compared to various clinical bladder cancer characteristics including grade (G), stage (T, N and M), tumor size, recurrence, progression, and cancer specific survival. The characteristics of the cancer patients and controls are shown in Table 1, and further comparisons were performed in separately categorized NMIBC and MIBC groups.

**Table 1:** Clinical and pathological features of patients with bladder cancer and controls

Variables	No. of patients (%)	No. of controls (%)
No.	305	34
Mean age ± SD	65.0 ± 12.6	53.8 ± 14.9
Gender		
Male	253 (83.0)	19 (55.9)
Female	52 (17.0)	15 (44.1)
Grade		
G1	56 (18.4)	
G2	132 (43.3)	
G3	117 (38.4)	
T stage		
Ta	48 (15.7)	
T1	136 (44.6)	
T2	55 (18.0)	
T3	38 (12.5)	
T4	28 (9.2)	
N stage		
N0	274 (89.8)	
N(1–3)	31 (10.2)	
M stage		
M0	287 (94.1)	
M1	18 (5.9)	
Median follow-up period (months)		

*SD* standard deviation

### The level of USP2a mRNA expression as a diagnostic marker for bladder cancer

*USP2a* mRNA expression in bladder cancer patients was significantly reduced (81.4 %) with respect to non-cancer patient controls (*p* < 0.001). The reduction in MIBC (*n* = 121) when compared to NMIBC (*n* = 184) was 36.3 % (*p* = 0.004) (Table 2). To further evaluate the reduction of USP2a during bladder cancer progression, we performed another qRT-PCR analysis using the second cohort, which is consisted with independent 140 patients (Table 3). BC tissues and adjacent normal bladder tissues from same patients were collected. In the cohort 2, USP2a mRNA expression of BC tissues was significantly decreased in comparison to matched surrounding normal mucosae (*p* < 0.001) (Table 3). The reduction of *USP2a* mRNA expression in cancerous tissues was 86.9 %, which was similar to that seen in the first cohort in comparison to healthy controls (Table 2).

**Table 2:** Levels of mRNA expression of *USP2a* were compared between bladder cancer and controls’ mucosae (Cohort 1)

Variables	Patients	mRNA expression of USP2a (median with IQR; × 103 copies/μl)	*p* value
Cancer vs. controls’ mucosae			<0.001
Controls	34	825.6 (370.1–1767.7)	
Cancer	305	153.9 (50.2–435.1)	
NMIBC vs. MIBC			0.004
NMIBC	184	180.2 (66.6–395.8)	
MIBC	121	114.8 (23.7–477.0)	

*IQR* interquartile range; *NMIBC* non-muscle invasive bladder cancer; *MIBC* muscle invasive bladder cancer

**Table 3:** Comparisons of *USP2a* expression and clinopathological features of bladder cancer (Cohort 2)

Variables	Patients	mRNA expression of USP2a (median with IQR; × 103 copies/μl)	*P* value
Cancer vs. matched surrounding tissue			<0.001
Surrounding tissue	140	1051.2 (403.4–2212.3)	
Cancer	140	138.1 (53.9–445.6)	

*IQR* interquartile range

### USP2a mRNA expression is significantly correlated to high stage of MIBC

With respect to several clinicopathological variables (e.g. age, sex, tumor size, number, grade, stage, and *USP2a* mRNA expression levels), low *USP2a* mRNA expression level was likely to reflect a significant risk of high stage (*p* = 0.024) in patients with MIBC, but not in NMIBC (Table 4). MIBC patients at higher stages such as T4 or N > 1 or M1 exhibited 52.5 % reduced *USP2a* expression, compared to MIBC patients at lower stages such as T2, T3, N0 or M0 (Table 4). However, no significant alteration of USP2a level was observed according to grades (Table 4). These data suggest that *USP2a* expression can be used specifically as a potential marker to stratify MIBC at higher stage.

**Table 4:** Comparisons of *USP2a* expression and clinopathological features of bladder cancer

Variable	Patient (%)	mRNA expression of *USP2a* (median with IQR; × 10^3 copies/μl)	*p* value
Grade			0.666
G1	56 (18.4)	186.4 (68.7–345.0)	
G2	132 (43.3)	151.4 (47.3–463.2)	
G3	117 (38.4)	137.3 (40.4–502.4)	
NMIBC			0.681
Ta	48 (26.1)	160.3 (68.7–340.2)	
T1	136 (73.9)	184.7 (65.4–489.5)	
MIBC			0.024
T2 or T3, N0, M0	67 (55.4)	136.7 (41.6–567.2)	
T4 or N ≥1 or M1	54 (44.6)	71.7 (15.8–296.1)	

*IQR* interquartile range; *NMIBC* non muscle invasive bladder cancer; *MIBC* muscle invasive bladder cancer

To test whether USP2a can be developed as a prognosis- or clinical outcome-related classifier, individual analyses were performed based on clinical outcomes (e.g. recurrence, progression, and overall survival). Analysis was performed with 118 NMIBC patients with no recurrence vs. 66 with recurrence, 162 NMIBC patients with no progression vs. 22 with progression, 38 MIBC patients with no progression vs. 83 with progression, and 49 alive MIBC patients vs. deceased 72 subjects (Table 5). Both in NMIBC and MIBC groups, we could not detect significant differences in *USP2a* expression when comparing recurrence (*p* = 0.756 for NMIBC), progression (*p* = 0.793 for NIBMC, and *p* = 0.912 for MIBC), and overall survival (*p* = 0.123 for MIBC).

**Table 5:** Comparisons of USP2a expression and clinical outcomes of bladder cancer patients

Variables	Patients (%)	mRNA expression of *USP2a* (median with IQR; × 10^3 copies/μl)	*p* value
NMIBC			
Recurrence			0.756
No recurrence	118 (64.1)	200.2 (77.9–391.7)	
Recurrence	66 (35.9)	157.6 (56.4–387.2)	
Progression			0.793
No progression	162 (88.0)	165.5 (65.0–355.3)	
Progression	22 (12.0)	238.9 (58.7–122.3)	
MIBC			
Progression			0.912
No progression	38 (31.4)	112.6 (23.1–508.7)	
Progression	83 (68.6)	115.2 (22.9–435.1)	
Overall survival			0.123
Alive	49 (40.5)	81.3 (16.1–483.3)	
Death	72 (59.5)	128.3 (37.4–494.1)	

*IQR* interquartile range; *NMIBC* non-muscle invasive bladder cancer; *MIBC* muscle invasive bladder cancer

Collectively, these data suggest that (1) *USP2a* level may be used for stratification of cancer from normal, or NMIBC from MIBC, (2) *USP2a* expression is not significantly associated with recurrence, progression or overall patient survival of NMIBC as well as MIBC.

## Discussion

Our results suggest that quantification of *USP2a* gene expression in bladder tumors may help differentiate normal vs. cancer, superficial vs. muscle invasive, and early MIBC vs. more advanced MIBC, when combined with traditional pathological testing and imaging. Analysis was conducted using 305 tissue specimens of bladder cancer patients with 34 age-matched controls, and 184 NMIBC and 121 MIBC patient tissues. A correlation between *USP2a* level and cancer is supported by analysis of a second cohort consisted of 140 bladder cancer specimens and their surrounding normal tissues. To our knowledge, this study presents the largest clinical data set describing *USP2a* gene expression in bladder cancer.

The patient-based results in this study suggest that *USP2a* expression was downregulated in bladder cancer tissues, which was inconsistent to previous in vitro studies implying the positive correlation of aggressiveness and *USP2a* [11, 12]. This discrepancies may be also explained by different and as yet poorly understood biological roles of USP2a, which is dependent on physiologic environment and cell context, or it may be the result of the natural composition of human specimens that include epithelial, stromal or inflammatory cells, and their communication with tumor cells.

There have been recent landmark studies defining the molecular phenotypes of bladder cancer, which may provide understanding the molecular and genetic events underlying BC progression. At least 3-4 molecular subtypes of BC were identified based on distinct mutations, and gene expression signatures, which may be connected to sensitivity to chemotherapy. The basal type BC subgroup expressing basal markers was dependent on the EGFR signaling pathway, thus this subgroup was significantly sensitive to treatment with drugs that inhibit the EGFR pathway. EGFR, a potential prognostic marker for MIBC has also been widely studied in bladder cancer [18]. Up-regulated EGFR signaling is known to initiate a cascade of events and lead to cell proliferation, migration, invasion and inhibition of apoptosis, all of which promotes tumor progression [23]. Altered expression of EGF family members (e.g. EGF, epiregulin and HB-EGF (heparin-binding epidermal growth factor-like growth factor)) and EGFR has been suggested as mediators of bladder cancer progression [24]. We showed previously that HB-EGF accumulates in the nucleus in aggressive TCC cells and is involved in an EGFR-dependent autocrine loop [24, 25], and that USP2a increases EGFR stability by inhibiting endocytosis and degradation of EGFR [10]. Based on the published literatures and the present data, *EGFR* and *USP2a* levels may be correlated with disease progression, although this has not been evaluated in the same series of tumors. A next step is to determine whether altered levels of *EGFR* [18] and *USP2a* are correlated with more aggressive bladder cancer, and whether these combined two molecular markers might predict aggressive disease.

Collectively, there is a critical need for methods that identify patients with MIBC that are likely to experience disease progression or metastasis. Our study demonstrated that *USP2a* expression levels make it possible to distinguish bladder cancer from normal, and muscle-invasive from non-invasive disease.

## Conclusions

The experimental results in this study provide an evidence suggesting that USP2a as a biomarker may improve diagnosis of human bladder cancer patients. Although the present analyses were performed with a large series of bladder cancer patients, which were carefully grouped into NMIBC vs. MIBC based on clinical evaluation, a larger prospective validation of our findings should be attempted.

## Abbreviations

NMIBC: Non-muscle invasive bladder cancer
MIBC: Muscle invasive bladder cancer
EGFR: Epidermal growth factor receptor
DUBs: Deubiquitinating enzymes
USP2a: Ubiquitin-specific protease 2a
FASN: Fatty acid synthase
OSCC: Oral squamous cell carcinoma
AJCC: American Joint Committee on Cancer
UHRF1: E3 ubiquitin-protein ligase UHRF1
PFKB: 6-phosphofructo-2-kinase/fructose-2,6-biphosphatase
KPNA2: Karyopherin-α2
GSTF6: Glutathione S-transferase 1
S100A8: S100 calcium binding protein A8
FGFR3: Fibroblast growth factor receptor-3
IL-1B: Interleukin-1 β
TUR: Transurethral resection
